# Development of a prediction model in female pure or predominant urge urinary incontinence: a retrospective cohort study

**DOI:** 10.1177/17562872221090319

**Published:** 2022-04-20

**Authors:** Tess van Doorn, Sarah H.M. Reuvers, Monique J. Roobol, Sebastiaan Remmers, Jan F.M. Verbeek, Jeroen R. Scheepe, Josien H. Wolterbeek, Deric K.E. van der Schoot, Daan Nieboer, Lisette A. ‘t Hoen, Bertil F.M. Blok

**Affiliations:** Department of Urology, Erasmus MC, Wytemaweg 80, Room Na 1524, 3015 CN Rotterdam, The Netherlands; Department of Urology, Erasmus MC Cancer Institute, University Medical Center Rotterdam, The Netherlands; Department of Urology, Erasmus MC Cancer Institute, University Medical Center Rotterdam, The Netherlands; Department of Urology, Erasmus MC Cancer Institute, University Medical Center Rotterdam, The Netherlands; Department of Urology, Erasmus MC Cancer Institute, University Medical Center Rotterdam, The Netherlands; Department of Urology, Erasmus MC Cancer Institute, University Medical Center Rotterdam, The Netherlands; Department of Urology, Franciscus Gasthuis & Vlietland, Rotterdam, The Netherlands; Department of Urology, Amphia Hospital, Breda, The Netherlands; Department of Urology, Erasmus MC Cancer Institute, University Medical Center Rotterdam, The NetherlandsDepartment of Public Health, Erasmus MC, Rotterdam, the Netherlands; Department of Urology, Erasmus MC Cancer Institute, University Medical Center Rotterdam, The Netherlands; Department of Urology, Erasmus MC Cancer Institute, University Medical Center Rotterdam, The Netherlands

**Keywords:** pelvic floor disorders, prediction model, treatment outcome, urinary incontinence, urinary urge incontinence

## Abstract

**Background:**

Urinary incontinence is a prevalent form of pelvic floor dysfunction, with a non-negligible impact on a patient’s quality of life. There are several treatment options, varying from conservative to invasive. The aim of this study is to predict treatment outcomes of pure or predominant urge urinary incontinence (UUI) in women to support shared decision-making and manage patient expectations.

**Methods:**

Data on patient characteristics, disease history, and investigations of 512 consecutive women treated for UUI in three hospitals in the Netherlands were retrospectively collected. The predicted outcome was the short-term subjective continence outcome, defined as patient-reported continence 3 months after treatment categorized as cure (no urinary leakage), improvement (any degree of improvement of urinary leakage), and failure (no improvement or worsening of urinary leakage). Multivariable ordinal regression with backward stepwise selection was performed to analyze association between outcome and patient’s characteristics. Interactions between patient characteristics and treatment were added to estimate individual treatment benefit. Discriminative ability was assessed with the ordinal c-statistic.

**Results:**

Conservative treatment was applied in 12% of the patients, pharmacological in 62%, and invasive in 26%. Subjective continence outcome was cure, improvement, and failure in 20%, 49%, and 31%, respectively. Number of incontinence episodes per day, voiding frequency during the day, subjective quantity of UI, coexistence of stress urinary incontinence (SUI), night incontinence, and bladder capacity and the interactions between these variables were included in the model. After internal validation, the ordinal c-statistic was 0.699.

**Conclusions:**

Six variables were of value to predict pure or predominant UUI treatment outcome in women. Further development into a comprehensive set of models for the use in various pelvic floor disorders and treatments is recommended to optimize individualized care. This model requires external validation before implementation in clinical practice.

## Introduction

Pelvic floor disorders (PFDs) such as urinary incontinence (UI) are highly common. Prevalence of UI varies between 13% and 50% for women^1^ and between 1% and 39% for men.^2^ Inevitably, UI has major impact on patients’ quality of life^3^ and has great economic impact because of high costs for treatment and absorbent products.^1^ Urge urinary incontinence (UUI), urinary leakage accompanied by a sudden compelling desire to pass urine,^4^ is one of the most common types of UI.^1^ Treatment options for UUI vary from conservative, like pelvic floor muscle training (PFMT) to minimal invasive therapy like sacral neuromodulation (SNM).^5,6^ UUI can occur in a pure form or in coexistence with stress urinary incontinence (SUI), which is defined by any involuntary urine loss on effort or physical exertion or on sneezing or coughing.^4^

Diagnosis and treatment decision-making in patients with PFDs is a complex and often subjective process, depending on the knowledge and preference of the caregiver and patient. This might result in suboptimal patient outcomes. A prediction model on different treatment outcomes can be used to provide support in the process of shared decision-making and to manage patients’ expectations.

A prediction model calculating the probability of the type of UI the patient suffers from and at the same time providing the optimal choice of treatment does not yet exist. Such a model may, if implemented into daily clinical practice, contribute to individualized care. An example of a successful multivariable prediction model is the Prostate Cancer Risk Calculator.^7^ This multiple externally validated prediction tool has shown to be able to reduce unnecessary testing in men with low risk of harboring a life-threatening prostate cancer.^7,8^

Such tools, combining diagnostics and prognostics, are currently scarce in the field of PFDs. As a first step in the development of a complete prediction model for PFDs in the future, we focus on pure or predominant UUI. In this study, we aim to develop a multivariable model to predict the effect on continence outcome after different UUI treatments. This model could aid in managing patients’ expectations and in shared decision-making when choosing UUI treatment in female patients, when implemented in clinical practice.

We hypothesized that specific predefined variables with univariate and multivariate analysis can predict the diagnosis and the outcome probability of various UUI treatments for individual patients on their characteristics. Examples of these variables are age, coexistence of SUI, and UI during the night. The total list of the predefined variables can be found in Table 1.

**Table 1.** Baseline patient characteristics – evaluated as potential predictors.

Characteristics	
Age (y)	60.8 (median, IQR 50.0–70.9)
Missing	0
Height	1.65 (median, IQR 1.60–1.70)
Missing	124 (24%)
Weight (kg)	75.0 (median, IQR 66.0–90.0)
Missing	125 (24%)
BMI, kg/m²	28.0 (median, IQR 24.5–32.1)
Missing	127 (25%)
Patient history	
Coexistence of SUI	
Yes	270 (52%)
No	196 (38%)
Missing	46 (9%)
In case of coexistence of SUI: predominant type of UI	
UUI	211 (41%)
SUI	19 (4%)
Equal	3 (1%)
No coexistence	196 (38%)
Missing	83 (16%)
Voiding frequency/24 h	13 (median, IQR 10–17)
Missing	226 (44%)
Voiding frequency during the day	10 (median, IQR 8–13)
Missing	209 (41%)
Voiding frequency during the night	3 (median, IQR 1–4)
Missing	193 (38%)
Incontinence pad use/24 h	3 (median, IQR 2–5)
Missing	121 (24%)
UI during night	
Yes	142 (28%)
No	82 (16%)
Missing	288 (56%)
Vaginal deliveries	
0	90 (18%)
1	68 (13%)
More than 1	273 (53%)
Missing	81 (16%)
Episiotomies or spontaneous lacerations (during vaginal deliveries)	
0	128 (25%)
1	83 (16%)
More than 1	66 (13%)
Missing	235 (46%)
Comorbidities	
DM	
Yes	69 (13%)
No	443 (87%)
Missing	0
Cardiovascular disease	
Yes	225 (44%)
No	287 (56%)
Missing	0
COPD	
Yes	34 (7%)
No	476 (93%)
Missing	2 (0%)
Psychiatric disorders and/or sexual abuse	
Yes	96 (19%)
No	69 (13%)
Missing	347 (68%)
Previous treatments	
Previous treatments for UUI	
None	183 (36%)
Conservative	114 (22%)
Pharmacological	152 (30%)
Invasive	63 (12%)
Missing	0
Previous surgical treatments for SUI	
Yes	73 (14%)
No	438 (86%)
Missing	1 (0%)
Previous other invasive therapies with influence on continence status	
Yes	184 (36%)
No	326 (64%)
Missing	1 (0%)
Current treatment	
Type of UUI treatment	64 (12%)
Conservative	317 (62%)
Pharmacological	131 (26%)
Invasive	0
Missing	
Bladder diary	
Number of UI episodes/24 h	5 (median, IQR 2–8)
Missing	220 (43%)
Subjective quantity of UI	
None	20 (4%)
Drops	62 (12%)
A splash	103 (20%)
A lot	156 (31%)
Missing	171 (33%)
Number of incontinence pad use/24 h	3 (median, IQR 2–5)
Missing	121 (24%)
Voiding frequency/24 h	11 (median, IQR 9–114)
Missing	118 (23%)
Voiding frequency during the day	9 (median, IQR 7–12)
Missing	118 (23%)
Voiding frequency during the night	2 (median, IQR 1–3)
Missing	118 (23%)
Maximum portion of urine	350 (median, IQR 250–500)
Missing	135 (26%)
Mean volume of a portion of urine	166 (median, IQR 121–210)
Missing	135 (26%)
Total voided volume/24 h	1800 (median, IQR 1273–2363)
Missing	138 (27%)
Fluid intake/24 h	1725 (median, IQR 1350–2150)
Missing	281 (55%)
Urodynamic study	
DO	
Yes	169 (33%)
No	93 (18%)
Missing	250 (49%)
In case of DO: (*n* = 169), DO from ml filling	166 (median, IQR 80–268)
Missing	14 (8%)
In case of DO: (*n* = 169), leakage during DO	
Yes	93 (55%)
No	66 (39%)
Missing	10 (6%)
Bladder capacity	350 (median, IQR 210–480)
Missing	253 (49%)
Cough-stress test	
Positive	64 (12%)
Negative	187 (37%)
Missing	261 (51%)
Other investigations	
Cough-stress test (separate test)	
Positive	96 (19%)
Negative	158 (31%)
Missing	258 (50%)

BMI, body mass index; COPD, chronic obstructive pulmonary disease; DM, diabetes mellitus; DO, detrusor overactivity, IQR, interquartile range; kg, kilogram; ml, milliliter; SUI, stress urinary incontinence; UI, urinary incontinence; UUI, urge urinary incontinence; y, year. Data are displayed as *n* (%) or median (IQR). Numbers do not add up to 512 patients because of missing data.

## Methods

### Study design and subjects

This retrospective cohort study was conducted in one academic hospital, Erasmus Medical Center in Rotterdam, and two nonacademic hospitals, the Amphia Hospital in Breda and the Haven Hospital in Rotterdam, the Netherlands. All data were retrospectively collected from the electronic patient files. Our study cohort included data from 2010, 2013, or 2015/2016 depending on electronic patient data availability in each participating center. The sample size was based on this availability. Eligible patients were identified based on ‘diagnosis treatment combination codes’ used for reimbursement of health care costs in the Netherlands. Only data on the first UUI treatment during the inclusion period were evaluated based on the intention-to-treat principle. Patients with bladder stones, bladder cancer, urinary tract infections, urinary catheters, (congenital) anatomical abnormalities of the urinary tract, neuro-urological dysfunction, symptomatic pelvic organ prolapse, and pregnant women were excluded. For quality control, 5% of all data entered into the study database was cross-checked with the patient file (JS, TN, IG). The medical ethics review board of the Erasmus MC reviewed and approved the study protocol (MEC-2016-103) with a waiver of informed consent.

### Predicted outcome parameter

The predicted outcome parameter was the short-term subjective continence outcome reported 3 months after initiation of UUI treatment, and was categorized as cure (no urinary leakage), improvement (any degree of improvement of urinary leakage), or failure (no improvement or worsening of urinary leakage). A time range between 1 week and 6 months was accepted as a 3-month period, depending on the evaluated treatment. The evaluated effect of percutaneous nerve evaluation on the experienced UUI after 1 week was considered acceptable for inclusion. An outcome 1 week after initiation of PFMT was not acceptable for inclusion because this treatment option needs more time to reach possible therapeutic effects.

### Predictive variables

Potential predictive variables for treatment were identified during a consensus meeting among the involved clinical and basic researchers and were based on the experience in clinical practice and relevant literature. These variables can be found in Table 1.

Other previous invasive treatments with influence on the continence status included therapies such as a hysterectomy or prolapse surgery. For variables derived from bladder diaries, the mean values for 2 days were used if available. Detrusor overactivity (DO) was defined as any involuntary detrusor muscle contraction during filling cystometry.^4^ The cystometric bladder capacity was defined as maximum filling during urodynamic studies (UDSs). Cough-stress tests were considered positive in case of any urinary leakage during coughing or Valsalva maneuver.

The type of UUI treatment was categorized as conservative, pharmacological, or invasive. Conservative treatment was defined as any treatment for UUI without the use of invasive or drug therapy, such as PFMT. Invasive treatment was defined as any treatment involving incision or puncture of the skin or mucosa, such as SNM or botulinum toxin-A (BTX-A) injections in the detrusor muscle.

### Data analysis

Descriptive statistics were used to summarize data. For all variables except age, diabetes mellitus (DM), cardiovascular disease, and type of UUI treatment, data were missing. These missing data (1–68%) were imputed using multiple imputations with the chained equations.^9^ Because of high percentage of missing values, we did not include the variable ‘in case of DO: from ml filling’ and ‘leakage during DO’ in further analyses.

Ordinal logistic regression was used to evaluate the association between each prognostic factor and the three outcomes: dry, improvement, and failure. As a first step, predictors were entered into univariable ordinal regression models. Significant predictors from the univariable regression based on the backward stepwise selection procedure using Wald selection criterion (*p* < 0.157)^10^ were entered into the multivariable ordinal regression model. Subsequently, the main effect model was augmented with interaction of the three treatment options. Statistical significance of interactions in main model was quantified by the *p* value of the overall Wald test statistic.^11^

The overall model performance was assessed with the ordinal c-statistic.^12^ The ordinal c-statistic indicates the probability of correctly classifying two patients into two randomly selected outcome categories. The model was internally validated using bootstrapping with 200 samples. Predictor effects were shrunken using the calibration slope at internal validation as heuristic shrinkage factor. Because of the treatment interaction, the predictive value of a single predictor cannot easily be interpreted and is visualized. All statistical analyses were performed using R version 3.4.2 (R Foundation, Vienna, Austria) with package rms and mice.^9^

## Results

Based on ‘diagnosis treatment combination codes’, 1395 consecutive female adult patients with UUI were identified. Twenty-five percent of these patients were not treated (for UUI) or treatment was not adjusted and 33% was excluded based on one or more of the exclusion criteria. In total, 598 female patients met the inclusion criteria. Data of 86 of 598 (14.4%) were excluded because no short-term subjective continence outcome could be determined from the patient file. Of the 512 remaining patients, 235 (45.9%) were recruited from the Erasmus MC, 204 (39.8%) from the Amphia Hospital, and 73 (14.3%) from the Haven Hospital.

Conservative management consisted of PFMT in 60 of 64 patients (Table 1). The remaining four patients were advised to adjust their alcohol and caffeine intake and their overall fluid intake or were advised to urinate at fixed intervals. Antimuscarinics were prescribed in 232 of 317 and selective beta-3 adrenoceptor agonists in 83 of 317 patients. Invasive therapy was SNM for 99 patients, BTX-A injections for 29 patients, and percutaneous tibial nerve stimulation (PTNS) for 3 patients. The subjective continence outcome was determined at a median of 87 days [interquartile range (IQR) 43–101] after initiation of therapy. Table 2 shows the subjective continence outcomes categorized by type of treatment.

**Table 2.** Subjective continence outcome categorized by treatment groups.

	Conservative (*N* = 64)	Pharmacological (*N* = 317)	Invasive (*N* = 131)	Total
Cure	7 (10.9%)	51 (16.1%)	46 (35.1%)	104 (20.3%)
Improvement	30 (46.9%)	165 (52.0%)	55 (42.0%)	250 (48.8%)
Failure	27 (42.2%)	101 (31.9%)	30 (22.9%)	158 (30.9%)

In univariable analyses, number of UI episodes per 24 h, voiding frequency during the day based on patient history, the presence of coexistence of SUI based on patient history, the predominant type of UI, the presence of UI during night, bladder capacity in ml, and subjective quantity of UI were found significant (Table 3).

**Table 3.** Factors associated with successful treatment for UUI – univariate analysis.

Variable	OR (95% CI)
Characteristics	
Age	0.92 (0.74–1.14)
Length	0.94 (0.72–1.22)
Weight	1.10 (0.87–1.39)
BMI	1.12 (0.88–1.43)
Patient history	
Coexistence of SUI (yes)	0.69 (0.50–0.96)*
In case of coexistence of SUI: predominant type of UI (SUI *versus* UUI and no coexistence)	0.24 (0.09–0.62)*
Voiding frequency/24 h	1.15 (0.92–1.43)
Voiding frequency during the day	1.18 (0.97–1.43)*
Voiding frequency during the night	0.89 (0.70–1.14)
Incontinence pad use/24 h	1.00 (0.84–1.17)
UI during night (yes)	1.44 (0.89–2.32)*
Vaginal deliveries (1 or more *versus* no)	1.08 (0.64–1.82)
Episiotomies or spontaneous lacerations (1 or more *versus* no)	0.91 (0.58–1.44)
Comorbidities	
Comorbidity, any (yes)	0.96 (0.69–1.33)
Sexual and/or physical abuse (yes)	1.54 (0.68–3.47)
Psychiatric diagnosis (yes)	1.23 (0.61–2.49)
Previous treatments	
Previous treatments for UUI	
None	Ref
Conservative	0.83 (0.53–1.29)
Pharmacological	0.77 (0.51–1.15)
Invasive	1.20 (0.70–2.07)
Previous surgical treatments for SUI (yes)	1.31 (0.81–2.11)
Previous other invasive therapies with influence on continence status (yes)	1.17 (0.83–1.65)
Current treatment	
Conservative	Ref
Pharmacological	1.53 (0.92–2.53)*
Invasive	3.30 (1.85–5.87)*
Bladder diary	
Number of UI episodes/24 h	0.77 (0.59–0.99)*
Subjective quantity of UI (a lot *versus* a little)	0.70 (0.49–0.99)*
Number of incontinence pad use/24 h	0.92 (0.78–1.09)
Voiding frequency/24 h	0.96 (0.78–1.18)
Voiding frequency during the day	1.01 (0.81–1.26)
Voiding frequency during the night	0.87 (0.73–1.06)
Maximum portion of urine	0.92 (0.66–1.29)
Mean volume of a portion of urine	0.91 (0.73–1.15)
Total voided volume/24 h	1.03 (0.66–1.60)
Fluid intake/24 h	0.99 (0.77–1.26)
Urodynamic study	
DO (yes)	0.90 (0.58–1.38)
DO from ml filling	0.86 (0.54–1.35)
Leakage during DO	0.84 (0.46–1.51)
Bladder capacity	1.41 (1.10–1.83)*
Cough-stress test (positive)	1.37 (0.93–2.04)
Other investigations	
Cough-stress test (positive)	1.10 (0.58–2.08)

BMI, body mass index; CI, confidence interval; DO, detrusor overactivity; SUI, stress urinary incontinence; UI, urinary incontinence; UUI, urge urinary incontinence. Odds ratios (ORs) for continuous variables are calculated between the first and third quartile. OR > 1 indicates an improved cure rate. Comorbidity is positive in presence of cardiovascular disease, chronic obstructive pulmonary disease, or diabetes mellitus disease. *Significant with *p* < 0.157.

Predominant type of UI was removed from the database as a predictive value because of limited cases in the conservative and invasive treatment groups. All other predictors were still significant after the backward selection procedure. Addition of treatment interaction resulted in an increase in model fit (Wald comparison test: *p* = 0.022). The ordinal c-statistic for the complete model was 0.732. After internal validation, the calibration slope was equal to 0.78 and the optimized corrected ordinal c-statistic was 0.699 [95% confidence interval (CI): 0.616–0.782].

**Figure 1.**
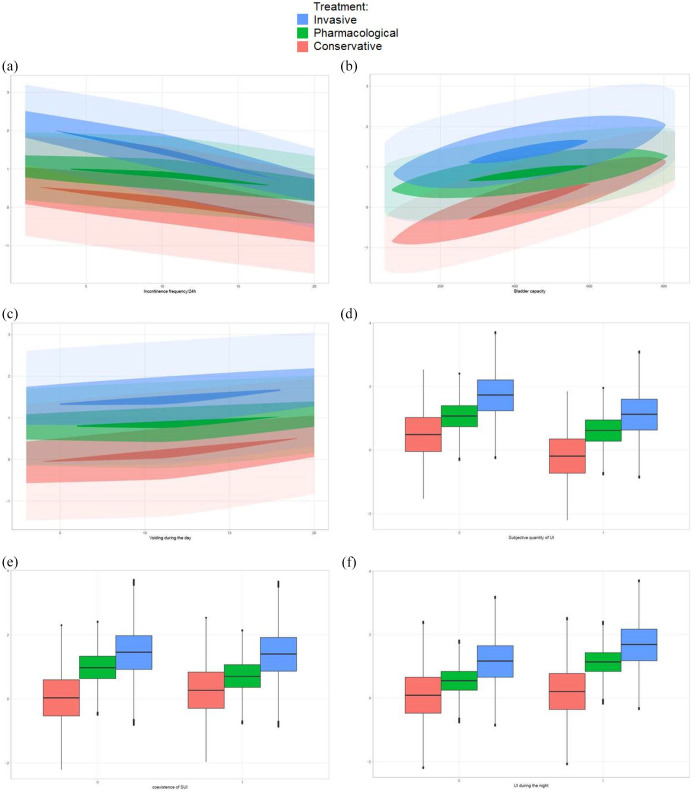
Predictive value of the six predictors visualized with density estimates and boxplots, stratified to treatment option. A higher logit indicates a higher probability of improvement or cure. The dark color to the lighter color enclose 5%, 50%, and 95% of all point’s estimates, respectively. (a) Predictive value of incontinence frequencies/24 h, (b) predictive value of bladder capacity, (c) predictive value of voiding during the day, (d) predictive value of subjective quantity of UI, (e) predictive value of coexistence of SUI, and (f) predictive value of UI during the night.

Figure 1, based on the average patient in our cohort, displays the predictors on the X-axis and the predictive ability (logit) on the Y-axis, where a higher logit indicates a higher probability of improvement or cure. In general, a larger bladder capacity, higher voiding frequency during the day, and UI during the night increase the probability of a successful treatment, whereas the subjective quantity of UI ranked as ‘a lot’, the coexistence of SUI, and an increased number of UI episodes/24 h decrease the probability of a successful treatment. The predicted outcome of pure or predominant UUI treatment for the several factors is also illustrated in two fictive patients in Table 4.

**Table 4.** The predicted short-term subjective continence outcome for two fictive patients based on our multivariate prediction model.

Patient information – model predictors		Predicted short-term subjective continence outcome
Female patient, 6 urinary incontinence episode/24 h, 11 voiding frequencies during the day, coexistence of SUI, no extensive subjective quantity of UI, UI during the night, and a bladder capacity of 400 cc.		
Type of treatment	Conservative	Probability of cure: 12% Probability of improvement: 52% Probability of failure: 36%
Type of treatment	Pharmacological	Probability of cure: 20% Probability of improvement: 56% Probability of failure: 24%
Type of treatment	Invasive	Probability of cure: 38% Probability of improvement: 51% Probability of failure: 11%
**Female patient, 15 urinary incontinence episode/24 h, 11 voiding frequencies during the day, coexistence of SUI, extensive subjective quantity of UI, no UI during the night, and a bladder capacity of 300 cc.**		
Type of treatment	Conservative	Probability of cure: 3% Probability of improvement: 27% Probability of failure: 70%
Type of treatment	Pharmacological	Probability of cure: 6% Probability of improvement: 39% Probability of failure: 55%
Type of treatment	Invasive	Probability of cure: 8% Probability of improvement: 45% Probability of failure: 47%

SUI, stress urinary incontinence; UI, urinary incontinence.

## Discussion

We developed a multivariable prediction model to estimate the effect of pure or predominant UUI treatment in female patients. The model, providing estimates of treatment outcome, includes information from bladder diaries, patient history, and UDSs. We found that the following aspects were of predictive value for treatment outcome: number of UI episodes per 24 hours, voiding frequency during the day based on patient history, the presence of coexistence of SUI based on patient history, the presence of UI during night, bladder capacity in ml, and subjective quantity of UI. In general, a larger bladder capacity, higher voiding frequency during the day, and UI during the night increase the probability of a successful treatment, whereas the subjective quantity of UI ranked as ‘a lot’, the coexistence of SUI, and an increased number of UI episodes/24 h decrease the probability of a successful treatment. In Table 4, the predicted short-term outcome of UUI treatment for the several factors is illustrated for two fictive patients.

Other prediction models in the field of PFDs and functional urology are, for example, a model that predicts the future risk of PFDs on the basis of high-risk characteristics during pregnancy.^13^ More relevant to the results of this study, Darekar *et al.*^14^ reported on the development of a model to predict the outcome of UUI treatment with fesoterodine. In both our studies and that of Darekar *et al.*,^14^ a lower number of UI episodes/24 h were found to be a positive predictor for treatment success. Herschorn *et al*.^15^ also acknowledged this variable as a predictor for treatment success after antimuscarinic treatment. Although using other outcome parameters (reduction in UI episodes or symptoms), Yazdany *et al.*^16^ and Richter *et al.*^17^ also found associations between the number of UI episodes/24 h and outcome of UUI therapies (BTX-A and SNM). It is shown in this study that the quantity of UI ranked as ‘a lot’ rather than ‘none or a little’ decreases the likelihood of treatment success.

The presence of UUI is often a symptom of the overactive bladder syndrome, defined as a complex of symptoms including urinary urgency, with or without UUI, usually with voiding frequency and nycturia.^4^ Patients with this syndrome often present clinically with high voiding frequency and small portions of urine. We had expected that a higher probability of cure after UUI treatment would be associated with a decrease in incontinence episodes and a higher bladder capacity, reflecting a lower severity overactive bladder syndrome. For example, a probability of treatment success of BTX-A was described to be associated with lower baseline score of overactive bladder symptoms.^18^ In line with this, in our study, we found that a positive treatment outcome was associated with a higher bladder capacity. We found that a positive treatment outcome was associated with a higher voiding frequency during the day before treatment. This can be explained by the rationale that a higher voiding frequency reflects a lower severity overactive bladder syndrome because a decreased amount of incontinence volume may result in an increased total voided volume and, therefore, in an increased voiding frequency during the day.

The coexistence of SUI and UUI is associated with worse outcomes in incontinence treatments such as PFMT and surgical treatments for SUI.^19^ In our study, the coexistence of SUI (based on patient history) was found to be a predicting factor for failure of treatments, especially in patients with predominant SUI. A possible explanation is that by treating the UUI component, the untreated SUI component might become more dominant. These results in patients with mixed UI could suggest that an integral therapy approach, including PFMT, drugs, and neuromodulation, should be considered. Fifty-two percent of patients included in the model had mixed UI, and the coexistence of SUI might have caused bias.

In addition, we found that patients who experienced UI during the night had a higher probability of cure and improvement. The presence of UI during the night might be indicative for the presence of UUI. A possible explanation could be that UI during the night might be more representative for pure UUI, less for a mixed form of UI.

Variables concerning DO derived from urodynamics were not found to be predictive for the outcome of UUI treatment. This is in concordance with findings from a systematic review of Rachaneni and Latthe^20^ that concluded that the presence of DO seems not to influence the effectiveness of invasive UUI treatments.

In this study, comorbidities and previous treatments were not found as predictors for outcome of treatment. The scarce evidence on this topic is contradictory. While Darekar *et al.*^14^ also did not find prior pharmacological UUI treatment and DM predictive for pharmacological treatment outcome, Herschorn *et al.*^15^ did find previous UUI treatments to be predictive. Khan *et al.*^21^ found that depression or anxiety might influence the outcome of PFMT; Marcelissen *et al.*^22^ found that psychological factors could not predict success in SNM. If future research confirms our findings, this might be valuable information for clinical decision-making. In current clinical practice, comorbidities and previous treatments are often a reason to refrain from further (invasive) treatments in these patients to avoid burdensome procedures.

Other bladder diary and patient history-derived parameters, such as the number of incontinence pad use/24 h, portions of urine, and voiding frequency during the night, were not found as predictors for UUI. We had expected that especially pad use would have predictive value, based on the idea that this reflects the severity of UUI. On the other hand, Dylewski *et al.*^23^ showed that the number of pad use is not associated with the quantity of urine lost.

The strength of this study lies in the model that predicts the improvement or cure not only of a single treatment but also of three UUI treatment outcomes based on multicenter data. This makes it possible to compare between the three treatment options and aids in shared decision-making. This study represents the first step of developing a comprehensive set of prediction models for PFDs. However, several limitations of our study need to be mentioned. First, bias could have been introduced because of the retrospective study design and treatment decisions being based on the judgment of the treating physician. This bias could be reduced in a randomized (collaborative) setting with increased sample size and prospectively collected data.^24^ Second, because of small numbers for specific treatments (e.g. BTX-A, SNM), treatment benefit could only be categorized as conservative, pharmacological, or invasive. It might well be that the predicting factors differ per specific treatment. Third, only internal validation could be performed and external validation is still required to confirm the model’s performance.^25^ Fourth, there were a high number of missing data, which were imputed; however, we cannot rule out that variables entering the main model were selected or rejected based on chance. Fifth, because of the retrospective study design, the outcome parameter was determined at a median of 87 days (IQR 43–101) after initiation of therapy and not for every patient at the ideal time of 3 months after initiation of the therapy. This variability might have influenced outcomes of our study. Finally, the choice of the outcome parameter ‘subjective continence outcome’ could be criticized. We chose however to include this outcome parameter because it is commonly used in clinical care. Disadvantages are the subjectivity of the measure, the lack of standardization, and the wide range of patients categorized under ‘improvement’. Ideally a validated questionnaire, with a previously determined minimally important clinical change, should serve as an outcome parameter. In contrast, from a clinical point of view, treatment for UI is based on patient-reporting and the subjective continence outcome is easy recognizable for patients in shared decision-making.

Our recommendation for future research is to externally validate this prediction model, so it can be used as an aid in shared decision-making for the use in daily practice and in managing patients’ expectations. External validation is preferred over internal bootstrapping (validation of the model in your own data) because of a high chance of bias. For this model, we included patients from academic and nonacademic hospitals, to develop a generalizable tool. We suggest to validate this tool in different centers, to cover the total patient population.

## Conclusions

We identified the following six independent predictors for the short-term subjective continence outcome of UUI treatments in women and developed a multivariable prognostic prediction model: number of UI episodes per 24 h, voiding frequency during the day based on patient history, the presence of coexistence of SUI based on patient history, the presence of UI during night, bladder capacity in ml, and subjective quantity of UI. The model has the potential to be used as an aid in decision-making for patients and physicians and in managing patients’ expectations in future perspective. This study represents a first step in developing prediction models for use in various PFDs. Before implementation of the prediction model in practice, external validation is required and further testing of the model in specific patient populations is recommended
